# Corrigendum

**DOI:** 10.2471/BLT.18.110418

**Published:** 2018-04-01

In: Sekhri N, Savedoff W. Private health insurance: implications for developing countries. Bull World Health Organ. 2005 February 1;83(2):127–34: 

on page 132, figure 4, South Africa’s private health insurance expenditure should read “42.4%”.

